# Primary Open Angle Glaucoma Is Associated With Functional Brain Network Reorganization

**DOI:** 10.3389/fneur.2019.01134

**Published:** 2019-10-25

**Authors:** Silvia Minosse, Francesco Garaci, Alessio Martucci, Simona Lanzafame, Francesca Di Giuliano, Eliseo Picchi, Massimo Cesareo, Raffaele Mancino, Maria Guerrisi, Chiara Adriana Pistolese, Roberto Floris, Carlo Nucci, Nicola Toschi

**Affiliations:** ^1^Department of Biomedicine and Prevention, University of Rome “Tor Vergata”, Rome, Italy; ^2^Neuroradiology Unit, Department of Biomedicine and Prevention, University of Rome “Tor Vergata”, Rome, Italy; ^3^San Raffaele Cassino, Cassino, Italy; ^4^Ophthalmology Unit, Department of Experimental Medicine, University of Rome Tor Vergata, Rome, Italy; ^5^Diagnostic Imaging Unit, Department of Biomedicine and Prevention, University of Rome Tor Vergata, Rome, Italy; ^6^Athinoula A. Martinos Center for Biomedical Imaging, Harvard Medical School, Boston, MA, United States

**Keywords:** resting-state functional magnetic resonance imaging (rs-fMRI), open angle glaucoma, graph theoretical measures, functional brain networks, neurodegenerative diseases

## Abstract

**Background:** Resting-state functional magnetic resonance imaging (rs-fMRI) is commonly employed to study changes in functional brain connectivity. The recent hypothesis of a brain involvement in primary open angle Glaucoma has sprung interest for neuroimaging studies in this classically ophthalmological pathology.

**Object:** We explored a putative reorganization of functional brain networks in Glaucomatous patients, and evaluated the potential of functional network disruption indices as biomarkers of disease severity in terms of their relationship to clinical variables as well as select retinal layer thicknesses.

**Methods:** Nineteen Glaucoma patients and 16 healthy control subjects (age: 50–76, mean 61.0 ± 8.2 years) underwent rs-fMRI examination at 3T. After preprocessing, rs-fMRI time series were parcellated into 116 regions using the Automated Anatomical Labeling atlas and adjacency matrices were computed based on partial correlations. Graph-theoretical measures of integration, segregation and centrality as well as group-wise and subject-wise disruption index estimates (which use regression of graph-theoretical metrics across subjects to quantify overall network changes) were then generated for all subjects. All subjects also underwent Optical Coherence Tomography (OCT) and visual field index (VFI) quantification. We then examined associations between brain network measures and VFI, as well as thickness of retinal nerve fiber layer (RNFL) and macular ganglion cell layer (MaculaGCL).

**Results:** In Glaucoma, group-wise disruption indices were negative for all graph theoretical metrics. Also, we found statistically significant group-wise differences in subject-wise disruption indexes in all local metrics. Two brain regions serving as hubs in healthy controls were not present in the Glaucoma group. Instead, three hub regions were present in Glaucoma patients but not in controls. We found significant associations between all disruption indices and VFI, RNFL as well as MaculaGCL. The disruption index based on the clustering coefficient yielded the best discriminative power for differentiating Glaucoma patients from healthy controls [Area Under the ROC curve (AUC) 0.91, sensitivity, 100%; specificity, 78.95%].

**Conclusions:** Our findings support a possible relationship between functional brain changes and disease severity in Glaucoma, as well as alternative explanations for motor and cognitive symptoms in Glaucoma, possibly pointing toward an inclusion of this pathology in the heterogeneous group of disconnection syndromes.

## Introduction

Glaucoma as one of the major causes of blindness in the world. Glaucoma is an optic neuropathy characterized by retinal ganglion cells death and degeneration of the optic nerve (1, 2). In this debilitating disease, any additional biomarker able to detect and quantify neuronal changes can aid in formulating better prognosis, monitor therapy outcomes and therefore influence quality of life (3).

Several diffusion tensor imaging (DTI) studies have demonstrated the involvement and degeneration of specific brain regions and white matter (WM) bundles in patients affected by Glaucoma (2, 4). Additionally, there is evidence of changes in the regional homogeneity and low frequency fluctuations in fMRI signals in Glaucoma patients compared to controls (5, 6). In this context, the recent hypothesis of brain involvement in pathologies of the visual system has sprung interest for neuroimaging studies in this realm, with a particular focus on primary open angle Glaucoma.

Overall, the mechanism underlying brain involvement in Glaucoma is hypothesized to be supported by a combination of both functional changes and structural damage. For example, a recent paper (7) demonstrated that connectivity between specific brain regions is associated with disease severity in as patients affected by Glaucoma, and that several structural brain abnormalities (as compared to healthy controls) can be detected in Glaucoma patients (4, 8, 9). Also, a global reorganization of brain networks in Glaucoma has been shown in a study (10) focused on cortical region and excluding the cerebellum. Interestingly, the latter region may be of particular interest in the study of brain involvement in glaucoma in view of the motor difficulties faced by Glaucoma patients (11, 12). Finally, a few studies examined the correlation between visual function tests outcomes and structural MRI findings in the anterior visual pathway in Glaucoma patients (13, 14), and an association between structural, functional and metabolic brain changes and optical coherence tomography (OCT) measures was recently shown (15).

Resting-state functional magnetic resonance imaging (rs-fMRI) is commonly employed to study changes in functional brain connectivity in a vast number of conditions, including neurodegenerative diseases such as Parkinson's or Alzheimer's disease. The interest in the so-called functional connectome (i.e., the complex network of cross-talks between brain areas) is ever increasing (16–18). To this end, recently several methods which stem from the realms of graph theory and network science have emerged as useful tools to study both local and global properties of complex brain networks. In detail, the brain is conceptualized as a graph, in which brain regions represent nodes and the relationships between the regions, defined through a variety of association measures fMRI time-series, represent edges which connect the nodes within the graph (19). Then, topological properties that highlight brain organization can be extracted (20). Recently, various studies have shown that graph-theoretical indices are sensitive to changes in brain network measures in both psychiatric and neurological diseases (16).

The purpose of this study was to evaluate a putative reorganization of functional brain networks in patients affected by primary open angle Glaucoma through graph- theoretical measures. To this end, we employ adjacency matrices based on partial correlation measures, in order to avoid the redundancies commonly introduced by the use of bivariate associations measures. Further, we exploit the recently introduced idea of a “disruption index” (21), which simultaneously takes global and local topological metric into account and allows to define the comparison between patients and controls in terms of how much the distribution of such measures is disrupted across the brain. Finally, in order to evaluate the potential of these disruption indices to serve as biomarkers to monitor disease severity, we explore their discriminative power between healthy and Glaucoma population as well as possible associations between functional brain reorganization indices, functional visual parameters, and thickness of select retinal layers measured through OCT.

## Materials and Methods

**Figure 1:**
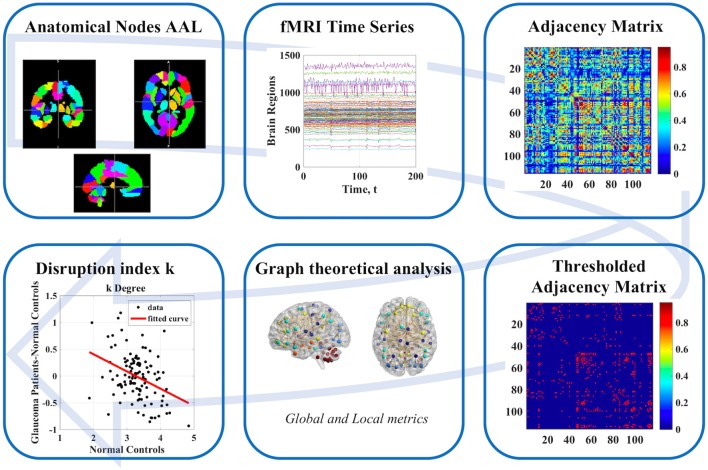
Schematic illustration of workflow from data to association matrix and graph analysis.

The overall workflow of our study is shown in Figure 1.

### Subjects

Nineteen Glaucoma patients and 16 healthy control subjects were enrolled from the Glaucoma Clinic and the General Outpatients clinic at the University Hospital “Policlinico Tor Vergata” (Rome, Italy). Patient characteristics are detailed in Table 1. The study protocol was approved by the local Institutional Review Board and adhered to the tenets of the Declaration of Helsinki. All subjects provided written informed consent.

**Table 1:** Clinical characteristics of our study population.

	**Glaucoma**	**Controls**
Group size	19	16
Age (years)		
Mean (range)	61.3 (50–72)	60.8 (50–76)
Sex (male/female)	8/11	11/5
IOP in treatment		
Mean (range)	15.89 (12–19)	15.44 (12–18)
Disease stage		
I	9	–
II	2	–
III	4	–
IV	1	–
V	3	–

*IOP, Intraocular pressure*.

After Glaucoma diagnosis, Glaucoma patients were eligible for the current study if they fulfilled the following inclusion criteria: (i) best corrected visual acuity > 0.1 logMAR, (ii) refractive error < ±5 spherical diopters or < ±3 cylindrical diopters, (iii) transparent ocular media, and (iv) open anterior chamber (Shaffer classification >20°). Exclusion criteria for Glaucoma patients as well as healthy controls were: (i) previous or active optic neuropathies, (ii) retinal vascular diseases, (iii) preproliferative or proliferative diabetic retinopathy, (iv) macular degeneration, (v) hereditary retinal dystrophy, (vi) use of medication that could affect Visual Field, (vii) previous or active neurological, cerebrovascular, or neurodegenerative diseases. Normal tension Glaucoma patients were also excluded (22). A Glaucoma diagnosis was defined following the European Glaucoma Society criteria (23). Glaucoma patients were treated using topical beta-blockers, prostaglandin analogs, and carbonic anhydrase inhibitors, alone or in fixed or unfixed combination.

### Ophthalmological Data Collection

After administering a medical history questionnaire, best-corrected visual acuity, anterior segment examination, intraocular pressure (IOP) measurement, ultrasound pachymetry, gonioscopy, and standard automated perimetry tests were administered to all subjects. Visual Field (VF) examination was performed using Humphrey Swedish Interactive Threshold Algorithm (SITA) standard with 24-2 test point pattern (Carl Zeiss Meditec Inc., Dublin, CA). The visual filed index (VFI) is a recently introduced summary parameter which is automatically calculated from the pattern deviation map values, in such a way that the central points of the VF have a larger impact as compared to peripheral points. The VFI ranges from 100% for a normal VF to 0% for a completely abolished VF (24). After pupillary dilation, slit-lamp fundus examination and spectral domain-optical coherence tomography (SD-OCT) using Glaucoma Module Premium Edition (GMPE) software (Heidelberg Retinal Engineering, Dossenheim, Germany) were assessed (22). The SD-OCT offers a tool for macular segmentation and thickness evaluation of individual retinal layers as well as Retinal Nerve Fiber Layer (RNFL) thickness. For each layer [macular total retina, Retinal Nerve Fiber Layer RNFL, Ganglion Cell Layer (GCL), Inner Plexiform Layer, Inner Nuclear Layer, Outer Plexiform Layer, Outer Nuclear Layer, Retinal Pigmented Epithelium, Inner Retinal Layers, and Outer Retinal Layers], thickness measurements of all sectors, as defined by the Early Treatment Diabetic Retinopathy Study scheme (temporal inner, superior inner, nasal inner, inferior inner, temporal outer, superior outer, nasal outer, and inferior outer), were employed.

### MR Imaging Protocol and Preprocessing

The maximum time interval between MRI and Ophthalmological data collection examinations was 1 week. MRI examinations were performed on a 3T scanner system (Achieva, Philips Medical Systems, Best, The Netherlands) with dedicated 8-channel sensitivity encoding (SENSE) head coil. All MRI examinations included rs-fMRI and three-dimensional T1-weighted, magnetization prepared rapid gradient echo (MPRAGE) images. rs-fMRI was performed using single-shot echo planar imaging (EPI) with the following parameters: acquisition and reconstruction voxel size 3.31 mm^3^, repetition time (TR) = 3,000 ms, echo time (TE) = 30 ms, flip angle = 80°, field of view (FOV) = 212 × 198 mm^2^, 200 volumes/subjects. The T1 weighted images (3D MPRAGE) were acquired with the following parameters: acquisition and reconstruction voxel size 1 × 1 × 1.2 mm^3^, TR = 500 (ms), TE = 50 (ms), flip angle = 8°, FOV = 256 × 240 mm^2^. rs-fMRI data was preprocessed in FLS v. 6.0 (25). The first three volumes were discarded to allow for scanner stabilization. Motion, distortion, and slice timing correction were performed in the FSL software suite. Finally, preprocessed functional scans were nonlinearly coregistered to standard space via the high-resolution T1 weighted MPRAGE image.

### Graph Theoretical Measures

Network nodes were defined by parcellation of the whole brain into 116 regions defined through the automated anatomical labeling (AAL) atlas. After parcellation, node- and subject-specific timeseries were extracted by voxel-wise averaging of the rs-fMRI signal in each region. Partial correlation between all 116 timeseries was used to generate subject-wise adjacency matrices. Subsequently range of density thresholds (from 5 to 40% in steps of 5%) were applied to these matrices. Given that the main results were seen to be robust to the threshold, the results were reported based on a sparsity value of 10%, as commonly adopted in brain network literature. We calculated the following graph-theoretical measures for each subject: two local nodal measures [degree and betweenness centrality (BC)], one functional integration measures (global efficiency), four measures of functional segregation (local efficiency, clustering coefficient, transitivity, and modularity), and one measure of resilience (assortativity). All metrics were calculated using the Brain Connectivity Toolbox (20).

### Disruption Indices

Local measures were analyzed through the disruption index *k* (10, 21), which measures the degree of overall reorganization of a specific property in the whole network. For network measure *NM*_*i*_, where i = (degree, betweenness centrality, local efficiency, clustering coefficient, and spectral measure of centrality), the disruption index *k* is defined [both for a group of subjects (Equation 2)] and for a single subject (Equation 1, see below for an example) through the following linear regressions across all nodes:

(1) $$\begin{array}{r} NM_{i,l}-\frac{1}{C}\overset{C}{\underset{j=1}{\sum }}NM_{i,j}={k_{i,l}^{0}+k}_{i,l}\cdot \frac{1}{C}\overset{C}{\underset{j=1}{\sum }}NM_{i,j}+\varepsilon_{l} \end{array}$$

(2) $$\begin{array}{r} \frac{1}{G}\overset{G}{\underset{j=1}{\sum }}{NM}_{i,j}-\frac{1}{C}\overset{C}{\underset{j=1}{\sum }}{NM}_{i,j}={k_{i}^{0}+k}_{i}\cdot \frac{1}{C}\overset{C}{\underset{j=1}{\sum }}{NM}_{i,j}+\varepsilon \end{array}$$

where *NM*_*i, l*_, *NM*_*i, j* = *C*_, *NM*_*i, j* = *P*_ are the *i*-th network measures for all subjects (l = C+G), control group (C) and Glaucoma patients (G), respectively. $NM_{i}\;\epsilon \;{\mathbb{R}}^{N}$, where *N* is the number of the node (1–116). *k*_*i, l*_ and *k*_*i*_ are the disruption indices relative to the *i*-th network measures for a single subject and for the Glaucoma patient group, respectively. $k_{i,l}^{0}$ and $k_{i}^{0}$are constant terms, ε_*l*_ and ε are the linear regression residues.

**Figure 2:**
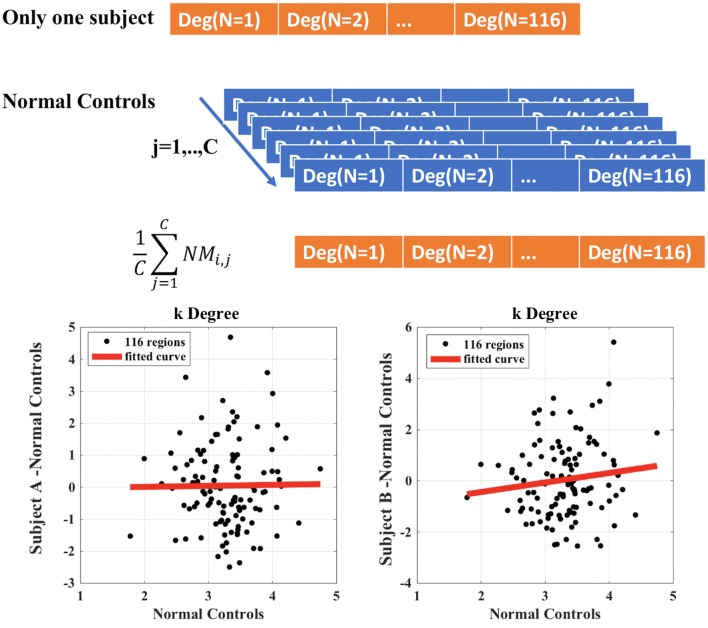
Example of calculation of the disruption index k for a single patient compared to control group. Subject A represents a healthy control patient; subject B represents a Glaucoma patient.

The calculation of *k*_*i, l*_ in the case of i = *degree*, subject A = control and subject b = Glaucoma patient is exemplified in Figure 2. The *y*-axis represents the difference between the degree of the single node of the subject (A or B) and the mean value of the degree of each node obtained in the control group. This latter quantity is also reported in the *x*-axis (mean value of the degree of each node obtained in the control group). The slope of the linear regression is the disruption index *k*. A disruption index *k* equal to zero implies that, on average, the degree of the node in a patient is close to the mean degree of the same node in a control group. If the disruption index *k* is statistically different from zero, the degree of the patient's node does not reflect the average degree of the same node in the control group, i.e., the degree of nodes in the network is completely reorganized (10, 21). The same rationale and algorithm can be applied to all other local network metrics.

### Network Hubs

In addition to calculating disruption indices, we identified subject-wise hub regions using all local network measures *NM*_*i*_ separately. For each subject, each region was classified as a hub when the respective network measure *NM*_*i*_ was at least 1.5 times higher than its whole-brain average.

### Measures of Brain Network and Clinical Parameters

The clinical parameters we employed in conjunction with graph-theoretical metrics are: (i) Retinal Nerve Fiber Layer (RNFL), (ii) Macula Ganglion Cell Layer (GCL), and (iii) Visual Field Index (VFI). The first two quantities were obtained as averages from OCT measures as follows:

$$\begin{array}{l} \bar{RNFL}=\;\frac{1}{6}\overset{6}{\underset{i=1}{\sum }}RNFL_{i}\\ \bar{MaculaGCL}=\;\frac{1}{9}\overset{9}{\underset{j=1}{\sum }}MaculaGCL_{j} \end{array}$$

where *i* = (temporal superior, nasal superior, nasal, nasal inferior, temporal inferior, temporal) and *j* = (Fovea, Temporal Inner, Superior Inner, Nasal Inner, Inferior Inner, Temporal Outer, Superior Outer, Nasal Outer, Inferior Outer). Both VFI and layer thicknesses were averaged across eyes for each patient. The left-right discrepancy between measures did not exceed 10% (VFI: mean 5.9 ± 8.8%; RNFL: mean 7.6 ± 6.7%, MaculaGCL: mean 3.2 ± 3.7%) in any of our patients.

### Statistical Analysis

Subject-wise global as well as local graph-theoretical metrics and subject-wise disruption indexes were compared statistically across groups using the nonparametric Mann–Whitney *U-*Test. For group-wise disruption indexes, we report the statistical significance of the overall regression. The interaction between the presence/absence of a hub at any specific node and group membership was tested using a Fisher's exact test. The association between functional brain measures (global and local graph-theoretical measures as well as disruption indices) and clinical parameters (*RNFL*, *MaculaGCL*, VFI) was assessed though separate linear regression models which included age and gender as nuisance covariates; for regression which yielded statistically significant results, Cohen's f^2^ was employed as a standardized measure of effect size. In the case of local measures, to exclude false positive results under multiple testing, a false discovery rate (FDR, alpha = 0.05) procedure was applied across all nodes (brain regions). *p* < 0.05 (FDR corrected) was considered statistically significant. In addition, receiver operating characteristic (ROC) analysis through binary logistic regression was performed in order to quantify the discrimination potential (between Glaucoma patients and healthy controls) of each metric and disruption index. The optimal operating point of each ROC curve was determined using Youden's index (which maximizes the sum of sensitivity and specificity), after which sensitivity, specificity, positive predicted value (PPV), and negative predicted value (NPV) were calculated. All data analysis was performed using in-house script written in MATLAB version 9.3.0, release 2017b (MathWorks, Natick, MA, USA).

## Results

**Figure 3:**
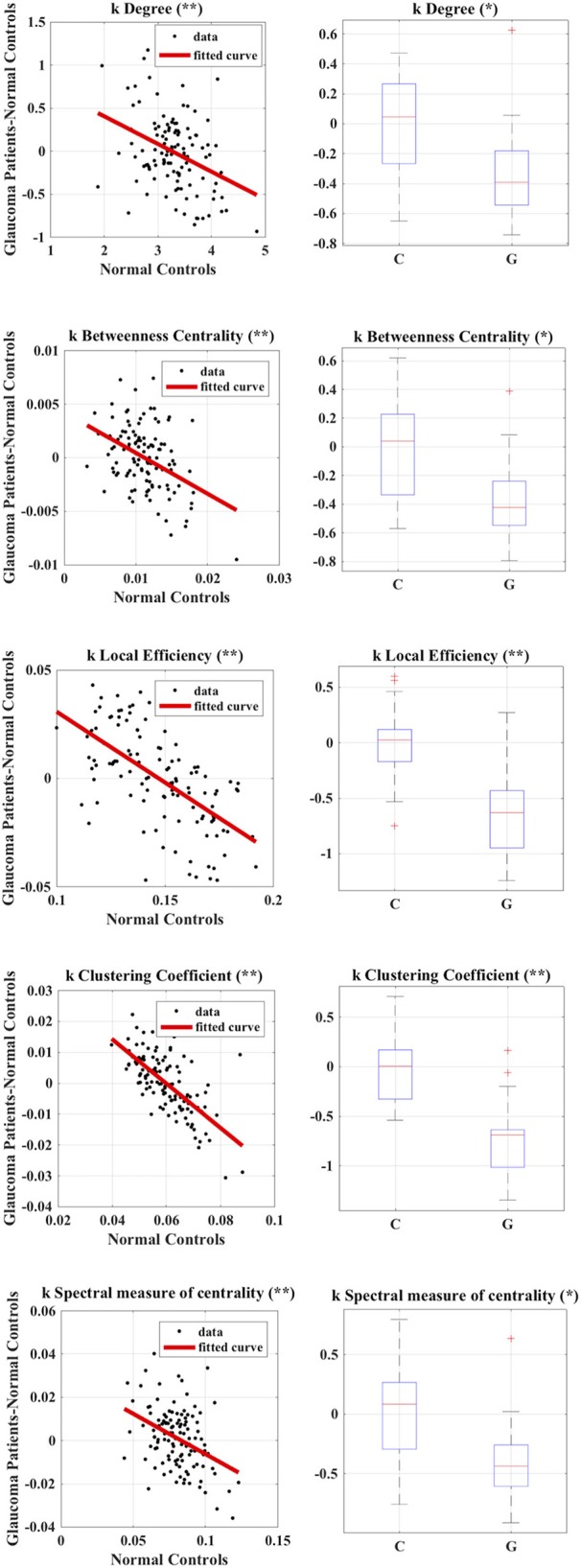
Calculation of group-wise disruption index **(left)** and group-wise differences in subject-wise disruption index *k* when comparing Glaucoma patients to healthy controls **(right)** in all local graph measures. *p < 0.05, **p < 0.001.

We found no statistically significant differences in age (*p* = 0.4, Mann–Whitney-*U*-Test) and gender (*p* = 0.5, Chi-Square160 Test) between the two groups. Table 2 summarizes the results obtained with group-wise disruption indices along with effect sizes (regression lines obtained while calculating the disruption indices are shown on the left of Figure 3). Figure 3 (right) also shows the results of group comparisons in subject-wise disruption indexes.

**Table 2:** Effect sizes (subject-wise disruption indices k) and regression slopes (group-wise disruption indices k).

**Network Measures**	**(Ki,C^Ki,G^)**	***p***	***k***	***p***
Degree	0.44	0.004	−0.32	<0.001
Betweenness centrality	0.46	0.004	−0.38	<0.001
Local efficiency	0.66	<0.001	−0.65	<0.001
Clustering coefficient	0.69	<0.001	−0.72	<0.001
Spectral centrality measure	0.52	0.006	−0.37	<0.001

*Effect size (second column from left): difference between the median values of the subject wise disruption indices along with p-values resulting from Mann–Whitney-U-tests (third column). Group-wise disruption indices k (fourth column from left) along with regression p-values (right column)*.

We found no statistically significant differences in global or local graph-theoretical metrics between Glaucoma and control patients. However, we found that all group-wise disruption indices were negative, and statistically different from 0 for all graph theoretical metrics (Figure 3 and Table 2). Additionally, statistically significant group-wise differences in subject-wise disruption indexes were found in all local metrics (Figure 3). For all statistically significant comparisons (i.e., in all disruption indices), the disruption index was lower in the Glaucoma group as compared to the healthy control group, highlighting a complex functional brain network reorganization pattern in Glaucoma patients.

**Figure 4:**
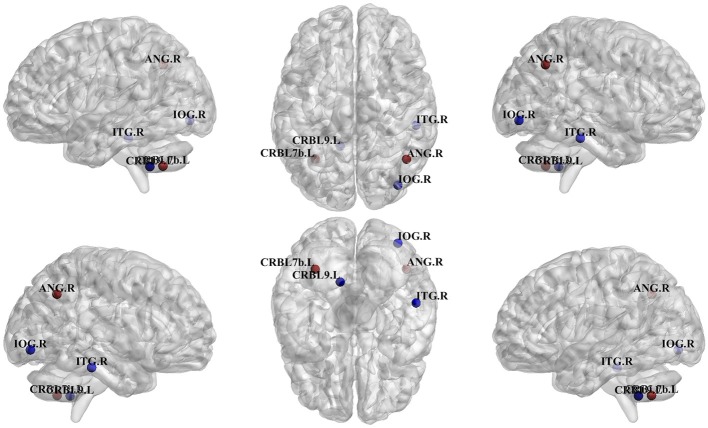
Differences in regions classified as hub in control vs. Glaucoma patients. The blue hubs “appear” and the red hubs “disappear” in Glaucoma patient as compared to controls. IOG.R, right inferior occipital; ANG.R, right angular gyrus; ITG.R, right inferior temporal gyrus; CRBL7b.L, left lobule VIIB of cerebellar hemisphere; CRBL9.L, left lobule IX of cerebellar hemisphere.

Figure 4 summarizes differences in regions which were classified as hubs between Glaucoma and control subjects. The left lobule VIIB of the cerebellar hemisphere (*p* = 0.035) was classified as a betweenness centrality hub in healthy controls but not in Glaucoma patients, and the right inferior occipital cortex (*p* = 0.010) behaved in the opposite manner. Also, we found that the right angular gyrus (*p* = 0.035) was classified as a spectral measure of centrality hub in healthy controls but not in Glaucoma patients, and that the right inferior temporal gyrus (*p* = 0.047) behaved in an opposite manner. Finally, the left lobule IX of cerebellar hemisphere (*p* = 0.047) was classified as a local efficiency hub in Glaucoma patients but not in healthy controls.

We found no statistically significant associations between global graph-theoretical metrics and clinical parameters. However, we found significant positive associations between disruption indices and VFI, *MaculaGCL* and *RNFL* (Table 3). When looking at associations between local graph-theoretical metrics and clinical parameters, significant positive associations were found in a number of regions, including the right parahippocampal gyrus, right transverse temporal gyrus and lobule X of vermis (Table 4).

**Table 3:** Results of linear regressions of disruption indices index k against clinical parameters.

**Network measures**	**VFI**			$\bar{\text{R}NF\text{L}}$			$\bar{\text{M}aculaGC\text{L}}$		
	**s**	**f^**2**^**	***p***	**s**	**f^**2**^**	***p***	**s**	**f^**2**^**	***p***
k Degree	+	0.059	0.129	+	0.207	0.023^*^	+	0.104	0.073
k Betweenness centrality	+	0.040	0.129	+	0.221	0.018^*^	+	0.122	0.047^*^
k Local efficiency	+	0.718	0.002^*^	+	1.140	<0.001^*^	+	0.993	0.001^*^
k Clustering coefficient	+	0.136	0.038^*^	+	0.657	<0.001^*^	+	0.454	0.002^*^
k Spectral centrality measure	+	0.060	0.108	+	0.192	0.023^*^	+	0.099	0.065

VFI, Visual Field Index; RNFL, Retinal Nerve Fiber Layer; Macula GCL, Macula Ganglion Cell Layer; s, sign of association; f^2^, Cohen's f^2^ (effect size); p, corrected significance level (FDR across 15 comparisons, alpha = 0.05); the asterisk

(*) * indicates statistically significant correlation (p < 0.05)*.

**Table 4:** Results of linear regressions of local graph-theoretical measures against clinical parameters.

**Regions**	**Network measures**	**VFI**			$\bar{\text{R}NF\text{L}}$			$\bar{\text{M}aculaGC\text{L}}$		
		**s**	**f^**2**^**	***p***	**s**	**f^**2**^**	***p***	**s**	**f^**2**^**	***p***
R parahippocampal gyrus	C	+	0.193	0.022^*^	+	0.171	0.023^*^	+	0.239	0.019^*^
R transverse temporal gyrus	Deg	+	0.142	0.048^*^			ns	+	0.206	0.022^*^
	El	+	0.118	0.046^*^			ns			ns
	v	+	0.158	0.046^*^	+	0.139	0.048^*^	+	0.277	0.014^*^
Lobule X of Vermis	C	+	0.208	0.049^*^			ns			ns

Abbreviations as in Table 3. L, left; R, right; El, Local Efficiency; C, Clustering Coefficient; BC, Betweenness Centrality; Deg, Degree; v, Spectral measure of centrality; ns, not statistically significant, s: sign of association, f^2^, Cohen's f^2^ (effect size); the asterisk (^*^) indicates statistically significant correlation (p < 0.05).

**Figure 5:**
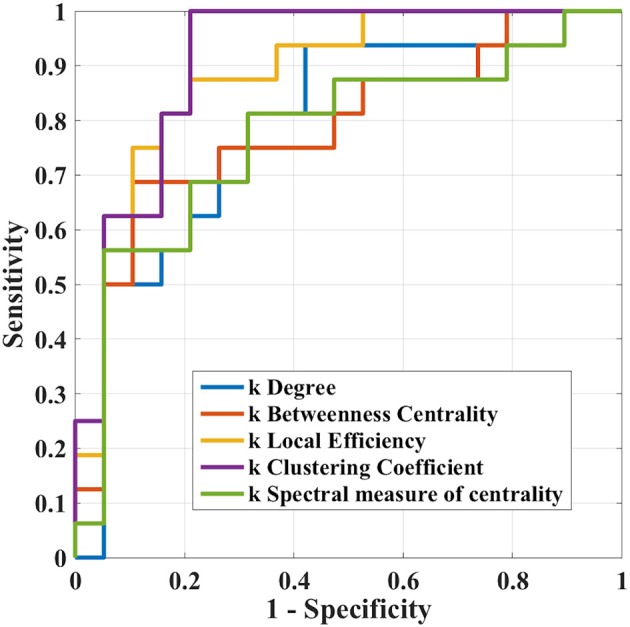
Receiver operation characteristic curves of *k*_*i*_ in the case where i = (clustering coefficient, local efficiency, degree, betweenness centrality, and spectral measure of centrality) in the differentiation task between Glaucoma patients and healthy controls.

The results of ROC analysis for disruption indices and global network measures are shown in Table 5. Overall, all disruption index yielded good to excellent (AUC = 0.773–0.911) discriminative power. The disruption index based on the clustering coefficient metrics yielded the best performance (AUC = 0.911, sensitivity = 100%; specificity = 78.95%) (Figure 5). Instead, global graph-theoretical metrics yielded fair to poor discriminative power. Finally, Table 6 shows the top 25 AUCs obtained when employed all single local graph-theoretical metrics as independent variables, which only yielded moderate discrimination performance (top AUC value = 0.72).

**Table 5:** Discrimination performance for global graph-theoretical metrics and disruption index for differentiating Glaucoma patients from healthy controls.

**Network measures**	**AUC**	**Sens (%)**	**Spec (%)**	**PPV (%)**	**NPV (%)**
k Clustering coefficient	0.911	100	78.95	80.00	100
k Local efficiency	0.885	87.50	78.95	77.78	88.24
k Degree	0.786	81.25	68.42	68.42	81.25
k Betweenness centrality	0.786	68.75	89.47	84.62	77.27
k Spectral measure of centrality	0.773	81.25	68.42	68.42	81.25
Betweenness centrality	0.582	87.50	42.11	56.00	80.00
Transitivity	0.549	62.50	52.63	52.63	62.50
Modularity	0.530	62.50	57.89	55.56	64.71
Clustering coefficient	0.520	87.50	36.84	53.85	77.78
Global efficiency	0.500	56.25	57.89	52.94	61.11
Degree	0.490	56.25	47.37	47.37	56.25
Assortativity	0.470	56.25	47.37	47.37	56.25
Eigenvector centrality	0.352	56.25	36.84	42.86	50.00

*k, disruption index; AUC, area under the receiver operating characteristic curve; Sens, sensitivity; Spec, specificity; PPV, positive predicted value; NPV, negative predicted value. AUC are ordered from high to low, top-down*.

**Table 6:** Discrimination performance for local graph-theoretical metrics for differentiating Glaucoma patients from healthy controls (top 25).

**Regions**	**Network measures**	**AUC**	**Sens (%)**	**Spec (%)**	**PPV (%)**	**NPV (%)**
L globus pallidus	BC	0.719	81.25	63.16	65.00	80.00
R parahippocampal gyrus	BC	0.712	68.75	73.68	68.75	73.68
L paracentral lobule	El	0.704	68.75	73.68	68.75	73.68
L supplementary motor area	SmC	0.701	81.25	63.16	65.00	80.00
L precuneus	BC	0.699	68.75	63.16	61.11	70.59
R middle frontal gyrus	SmC	0.697	75.00	78.95	75.00	78.95
R cuneus	El	0.697	62.50	89.47	83.33	73.91
L superior occipital gyrus	C	0.697	68.75	63.16	61.11	70.59
Rsupramarginal gyrus	El	0.694	62.50	73.68	66.67	70.00
L globus pallidus	SmC	0.691	68.75	68.42	64.71	72.22
L supplementary motor area	Deg	0.688	62.50	78.95	71.43	71.43
R parahippocampal gyrus	Deg	0.688	68.75	63.16	61.11	70.59
R supramarginal gyrus	Deg	0.688	56.25	68.42	60.00	65.00
R supplementary motor area	C	0.686	81.25	68.42	68.42	81.25
L lobule VI of cerebellar hemisphere	BC	0.684	68.75	63.16	61.11	70.59
R caudate nucleus	BC	0.679	75.00	68.42	66.67	76.47
L olfactory cortex	C	0.676	68.75	73.68	68.75	73.68
R lobule IV, V of cerebellar hemisphere	BC	0.676	56.25	84.21	75.00	69.57
R superior occipital gyrus	C	0.674	68.75	68.42	64.71	72.22
L inferior occipital	Deg	0.674	62.50	78.95	71.43	71.43
L inferior occipital	El	0.674	81.25	57.89	61.90	78.57
R supramarginal gyrus	BC	0.674	68.75	63.16	61.11	70.59
L transverse temporal gyrus	El	0.674	56.25	73.68	64.29	66.67
R superior parietal lobule	SmC	0.671	68.75	73.68	68.75	73.68
L globus pallidus	Deg	0.671	81.25	63.16	65.00	80.00

*Abbreviations as in Table 5. L, left; R, right; BC, Betweenness Centrality; El, Local Efficiency; SmC, Spectral measure of centrality; C, Clustering Coefficient; Deg, Degree*.

## Discussion

Advanced neuroimaging techniques have very recently begun to be employed to study the structural, functional, and metabolic changes in Glaucoma patients, including damage of gray matter atrophy and loss of structural connectivity (4), functional connectivity changes (10), and metabolite concentration (26). In this context, the involvement in Glaucoma of brain areas not directly responsible for the processing of visual information is beginning to emerge (4, 7, 9, 27).

In this study, we used advanced graph theoretical methods, including the recently defined idea of subject-wise and group-wise disruption index, to analyze the topological properties of brain connectivity in patients affected by Glaucoma. Our results provide novel insights into subtle functional alterations in the brain of Glaucoma patients, also extending recent findings on functional brain network reorganization in Glaucoma (10). As opposed to Wang et al. (10), in the present study we included cerebellar regions, an extensive array of graph-theoretical measures and used a non-redundant, fully multivariate associations measure to construct adjacency matrices, and used three different graph metrics (betweenness centrality, local efficiency and a spectral measure of centrality) to identify hubs, and analyzed correlations with the Visual Field Index, as opposed to the VFI MD. These multiple methodological differences may explain minor discrepancies between our findings and the results reported in Wang et al. (10). We found a profound whole-brain functional reorganization in Glaucomatous patients (all disruption indices were significantly lower in the Glaucoma group as compared to healthy controls) which was also reflected in network disruption and appearance-disappearance of specific hubs as compared to healthy controls. This in in keeping with a recently highlighted extensive brain dysfunction with and showed different spatial distribution in short- and long-range functional connectivity density in Glaucoma (28). ROC analysis confirmed that disruption indices yield remarkably high sensitivity and specificity and are therefore particularly useful in discriminating Glaucoma patients from healthy controls, hence candidating such indices as biomarkers for monitoring brain involvement and reorganization in Glaucoma. Their robust positive association with VFI and retinal thickness values further corroborates this possibility.

Two hub regions present in healthy controls “disappeared” in Glaucoma patients as compared to controls: (A) the right angular gyrus (which was classified as a spectral measure of centrality hub in healthy controls only, but not in Glaucoma patients). This region, located in the anterolateral region of parietal lobe, plays an important role in processing concepts rather than percepts when interfacing perception-to-recognition-to-action (29), possibly offering an alternative, non-mutually exclusive explanation (in addition to impaired vision) for the difficulty in distinguishing faces documented in Glaucoma patients (12); (B) The left lobule VIIB of cerebellar hemisphere (which was classified as a spectral measure of centrality hub in healthy controls only, but not in Glaucoma patients) plays an important role in fine motor coordination, in particular in the inhibition of involuntary movement via inhibitory neurotransmitters (30). Importantly, this could provide an alternative explanation (other than impaired vision) for the motor disturbances experienced by Glaucoma patients (12).

In contrast, three hubs were present in Glaucoma patients only (but not in healthy controls): (A) the right inferior occipital cortex (betweenness centrality hub): this region is located in the occipital lobe, which contains the primary visual pathway (15); (B) the right inferior temporal gyrus (spectral measure of centrality hub), this regions is located in the temporal lobe and has been found to be a key area in terms of simple processing of the visual field (31); (C) the left lobule IX of the cerebellar hemisphere (local efficiency hub); this area is considered essential for the visual guidance of movement (32). In this context, the first cortical transmission and processing station of the visual pathway is the primary visual cortex, from which information is transmitted to the parietal lobe and temporal lobe. There, information is processed and feedback is provided to the primary visual cortex. Given that the hubs not present in Glaucoma patients (i.e., in the parietal lobe and cerebellum) belong to secondary visual pathways, and that the hubs present only in Glaucoma patients are located in the occipital lobe, this reorganization could be hypothesized to reflect a complex interplay between neurodegeneration and functional compensatory mechanisms. In addition, our findings are not limited to the primary visual pathway. This is in agreement with previous structural (2, 4, 33) and functional imaging studies, which also highlight changes in brain areas related to working memory and attention in Glaucoma patients (4, 7, 9). Also, the fact that out of there three hubs, two were localized in the right hemisphere, may lend itself to a lateralization hypothesis, which should however be tested statistically in a larger patient sample.

Finally, while several studies have investigated associations between structural, functional, and metabolic brain measures and clinical parameters such as RNFL thickness and VFI (14, 15), such associations have not yet been studies through local and global disruption indices. Indeed, indices of brain network reorganization were significantly and positively related to VFI as well as structural retinal layer thicknesses. In addition, select local (as opposed to global) graph measures were positively related to VFI as well as structural retinal layer thicknesses. This points toward a direct link between the extent in functional rearrangement in both visual and extra-visual areas and both functional vision parameters (e.g., VFI) as well as structural indicators of disease severity (retinal thickness values), further corroborating the role of such disruption indices as possible biomarkers in Glaucoma. It should be noted that, since this is an associational and cross-sectional study, no definite inference is possible about the causality of the interactions we observed between visual impairments and functional brain reorganization. Indeed, altered functional connectivity of the primary visual cortex has been demonstrated in early and late blindness (34, 35). However, the fact that our findings involve not only primary, but also secondary visual regions could lead to speculate about a putative role of the latter secondary regions as contributors to the pathogenesis of Glaucoma. Taken together, our data highlight cerebral reorganization of brain networks in Glaucoma patients (36–39) supporting the interpretation of Glaucoma as central nervous system disease, likely part of the heterogeneous group of recently described disconnection syndromes (40). However, it should be noted that the number of patients assessed qualifies this work as an exploratory study, and that the optimal disruption index cut-off values estimated in this study may vary between centers due to e.g., differences in rs-fMRI acquisition protocols. Also, our experimental protocol did not include neurocognitive testing—we therefore cannot examine the putative associations between neurocognitive status and MRI parameters. Also, given that our study was performed in a relatively small sample size, future multicentric investigations in a larger number of patients and with longitudinal observations are warranted to precisely evaluate the true direction of the putative causal relationships between visual and brain manifestations of Glaucoma, and to quantify the potential of brain disruption indices as sensitive biomarkers of disease progression and brain involvement in this disease. Also, our patients were treated using topical beta-blockers, prostaglandin analogs and carbonic anhydrase inhibitors, alone or in fixed or unfixed combination. A recent study assessing glaucoma patients using resting state f-MRI reports that the possible subtle impact of these medications on intrinsic brain dynamics are not yet determined (41). Also, another study on patients treated with a beta-blocker or a prostaglandin analog reported that macular thickness, measured using OCT, did not to vary significantly both between the two groups and within each group during the 6-month evaluation (42). It is therefore likely that drug treatment ahs significantly interfered with our findings.

In summary, our data lend further support to the involvement of the central nervous system in Glaucoma supporting the hypothesis that glaucoma is a neurodegenerative disease. From the clinical point of view, this supports the usefulness of neuroprotective strategies in the treatment of glaucoma in association to the standard hypotensive treatments (36, 43–47).

## Data Availability Statement

The datasets for this manuscript are not publicly available because the data was acquired in our institution and is not available online. Requests to access the datasets should be directed to SM (silvia.minosse2@gmail.com).

## Ethics Statement

The study protocol was approved by the local Institutional Review Board and adhered to the tenets of the Declaration of Helsinki. All subjects provided written informed consent.

## Author Contributions

FG, CN, and NT managed the overall project (conceptualization, methodology, interpretation). SM and NT performed data preprocessing, statistical analysis, results interpretation, and prepared the original manuscript draft. FG, SL, FD, EP, MG, CP, and RF performed MRI data acquisition and database maintenance. AM, MC, RM, and CN were responsible for recruitment and ophthalmological examinations and results interpretation. All authors critically reviewed, read, and approved the submitted version of the manuscript.

### Conflict of Interest

The authors declare that the research was conducted in the absence of any commercial or financial relationships that could be construed as a potential conflict of interest.
